# Antioxidant Activity In Vitro Guided Screening and Identification of Flavonoids Antioxidants in the Extract from *Tetrastigma hemsleyanum* Diels et Gilg

**DOI:** 10.1155/2021/7195125

**Published:** 2021-11-23

**Authors:** Li Ding, Xiaomin Zhang, Jiajia Zhang

**Affiliations:** Zhejiang Pharmaceutical College, Ningbo, Zhejiang, China

## Abstract

This study aimed to investigate the extract with high antioxidant activity of *Tetrastigma hemsleyanum* Diels et Gilg and identify the antioxidant components in vitro. *α*, *α*-Diphenyl-*β*-picrylhydrazyl (DPPH) radical assay, Trolox equivalent antioxidant capacity (TEAC) assay, ferric reducing antioxidant power (FRAP), and hydroxyl radical scavenging method were used to screen the extract with high antioxidant activity. The antioxidant capacity of the extracts was evaluated by the free radical scavenging ability of DPPH. The ability of extracts to scavenge 2, 2'-azino-bis(3-ethylbenzothiazoline-6-sulfonic acid) (ABTS) free radical was evaluated by TEAC assay. The FRAP method was used to evaluate the ability of extracts to reduce Fe^3+^. The ability to scavenge hydroxyl radicals produced by the interaction of hydrogen peroxide and Fe^2+^ was measured by monitoring the change in the absorbance of the reaction mixture at 536 nm. Then, high-performance liquid chromatography-DPPH (HPLC-DPPH) and HPLC-hydroxyl radical scavenging methods were used to screen the antioxidant components in the extract. The molecular weight of the above antioxidant components was investigated using the qualitative analytical method of high-performance liquid chromatography coupled with quadrupole time-of-flight tandem mass spectrometry (HPLC-Q-TOF LC/MS). Based on the concentrations of the samples (0.2–4 mg/mL), the DPPH free radical scavenging ability, ABTS+ free radical scavenging ability, hydroxyl free radical scavenging ability, and Fe^3+^ reducing ability of the ethyl acetate extract (EAE) were stronger than that of the crude extract (CE), petroleum ether extract (PEE), and n-butanol extract (BE). The EAE has higher antioxidant activity than CE, PEE, and BE. Six antioxidant components, rutin, quercetin, isoquercetin, astragalin, kaempferol, and kaempferol-3-o-rutoside, were identified in the EAE.

## 1. Introduction

The rhizome of *Tetrastigma hemsleyanum* Diels et Gilg, a Chinese folk herbal medicine, is used for the treatment of children with high fever, pneumonia, hepatitis, viral meningitis, scrofula, carbuncle, and furuncle [1]. Modern pharmacological studies have shown that *Tetrastigma hemsleyanum* Diels et Gilg has anti-inflammatory, analgesic [2], antiviral [3], antiliver injury [4, 5], antitumor [6, 7], and other pharmacological effects [8, 9].

A large number of studies are focused on the chemical constituents of *Tetrastigma hemsleyanum* Diels et Gilg. The aboveground part of *Tetrastigma hemsleyanum* Diels et Gilg contains *β*-sitosterol, palmitic acid, pentacosane, gallic acid, ethyl gallate, catechin, 7-O-galloylcatechin, and 3,3'- dimethoxy ellagic acid-4-O-β-D-glucopyranoside [10]. Taraxerone, ergosterol, oleic acid, palmitic acid, myristic acid, hexadecylic acid, heptadecanoic acid, 9,12-octadecadienoic acid, *α*-linolenic acid, kaempferol, quercetin, and kaempferol-3-o-neohesperidin were found in liposolubility extract of *Tetrastigma hemsleyanum* Diels et Gilg [11–13]. In addition, Xu analyzed 80% methanol ultrasonic extract of *Tetrastigma hemsleyanum* by HPLC-Q-TOF-MS and found that it contained kaempferol-7-o-rhamnose-3-o-glucoside, rutin, isoquercetin, kaempferol rutoside, astragaloside, and quercetin [12]. Fan identified 8 flavonoids (isoorientin, orientin, orientin-2”-O-rhamnoside, isoorientin-2”-O-rhamnoside, vitexin, vitexin-2”-O-rhamnoside, isovitexin, and isovitexin-2”-O-rhamnoside) in *Tetrastigma hemsleyanum* leaves by rapid resolution liquid chromatography coupled with quadrupole time-of-flight tandem mass spectrometry (RRLC-Q-TOF-MS) [14]. These results showed that oleic acid and flavonoids constituted the largest proportion of the plant [15]. Flavonoids have various biological activities, such as the cardiovascular system [16, 17], antioxidant [18–20], anti-tumor [21–23], anti-inflammatory, and analgesic [24], and are one of the most and diverse phenolic groups of natural origin [25]; their antioxidation capability is a significant property.

Free radicals that are produced in the process of life metabolism regulate the signal transmission between cells and cell growth and inhibit the viruses and bacteria [26]. Excessive free radicals attack the cell membrane and leak from the intracellular region, which in turn leads to the denaturation of protein and nucleic acid, thereby, damaging the cells, tissues, and organs, inducing various diseases, and accelerating the aging of the body. The mechanism of inflammation, tumor, aging, and cardiovascular diseases is closely related to the excessive production of free radicals or a decrease in the ability to scavenge free radicals [27]. Antioxidants quench free radicals and form nontoxic ions or molecules. The free radicals were scavenged to achieve antioxidation, and antioxidants can stop or reverse some damage caused by excessive free radicals [28]. Synthetic antioxidants used to prolong the shelf life of food, such as butyl hydroxyanisole (BHA), dibutyl hydroxytoluene (BHT), and propyl gallate (PG) [29–31], have been found to be potential hepatotoxicity and carcinogenicity [32, 33]. Due to restricted use of synthetic antioxidants [34], natural antioxidants with high safety and fewer side effects are favored. Natural antioxidants and active substances mainly originate from plants [31], including tea extract [35, 36], fruit and vegetable extracts [37], balsam pear extracts [38], Chinese herbal extracts [39], raw Rehmanniae extract [40], and spices extracts [41]. Natural antioxidants can eliminate free radicals [42, 43], improve blood circulation, delay cell aging, and prevent cancer [44, 45].

While screening the antitumor extract and active components of *Tetrastigma hemsleyanum* Diels et Gilg, we found that some of the isolated parts were oxidized [46, 47]. This phenomenon indicated that there might be antioxidant components in *Tetrastigma hemsleyanum* Diels et Gilg. However, active components responsible for antioxidants in *Tetrastigma hemsleyanum* Diels et Gilg are yet to be identified. Therefore, by utilizing the antioxidant test, we screened out the extract that harbors high antioxidant activity, and the molecular weight of the potential active components was determined by HPLC-Q-TOF LC/MS.

## 2. Materials and Methods

### 2.1. Chemicals and Plant Material

DPPH, ABTS, TPTZ (2,4,6-tri-2-pyridinyl-1,3,5-triazine), and Trolox (6-hydroxy-2,5,7,8-tetramethylchroman-2-carboxylic acid) were purchased from Sigma (St. Louis, MO, USA), and methanol, ethanol, petroleum ether, ethyl acetate, n-butanol, and other reagents for the antioxidant test were analytically pure and purchased from China National Pharmaceutical Group Co. (Beijing, China). Rutin (lot no. 100080–201805, HPLC ≥98%), astragaloside (lot no. 110781–201908, HPLC ≥98%), isoquercetin (lot no. 111809–201804, HPLC ≥98%), quercetin (lot no. 100081–201610, HPLC ≥98%), kaempferol (lot no. 110861–201703, HPLC ≥98%), and kaempferol-3-o-rutoside (lot no. 112007–201903, HPLC ≥98%) standard reference materials were obtained from National Institutes for Food and Drug Control (Beijing, China). Chromatography grade and LC-MS grade acetonitrile were obtained from Tedia (Fairfield, OH, USA) and Fisher (Waltham, MA, USA), respectively.

The rhizome of *Tetrastigma hemsleyanum* Diels et Gilg from Guangxi Province, China, was purchased from Guangxi, China (lot no. 180615), and identified by Professor Xia Miao-fen at Zhejiang Pharmaceutical College.

### 2.2. Extraction, Fractionation, and Antioxidant Evaluation

The extracts were prepared according to the method of Bao et al. [48] and Yong [49] with minor modifications. The dried powder (5 kg) of the rhizomes of *Tetrastigma hemsleyanum* Diels et Gilg was soaked in three volumes of 60% methanol at room temperature. Subsequently, the soaking solution was filtered, combined, and concentrated under reduced pressure; the crude extract (CE) (364 g) was prepared by vacuum drying. Then, it was suspended in water and extracted successively with petroleum ether, ethyl acetate, and n-butanol to obtain petroleum ether extract (PEE) (28 g), ethyl acetate extract (EAE) (25 g), and n-butanol extract (BE) (68 g), respectively. The antioxidant activities of each extract were measured using the DPPH assay, TEAC assay, ferric reducing antioxidant power (FRAP), and hydroxyl radical scavenging method to screen the extract with high antioxidant activity. The antioxidant components in this extract were detected by HPLC-DPPH and HPLC-hydroxyl radical scavenging methods. The molecular weight of the antioxidant components was deduced by HPLC-Q-TOF LC/MS. The extracts were solubilized in ethanol solution (80% v/v), except for PEE, which was dissolved in dimethyl sulfoxide (DMSO).

### 2.3. DPPH Assay

The DPPH free radical scavenging activity was assessed according to Leong et al. [50] with a slight improvement. Briefly, 3.0 mL of DPPH 80% ethanol solution (10^−4^ M) was mixed with 0.1 mL of the sample solution. After incubation for 30 min in the dark at room temperature, the absorbance was measured at 517 nm using a UV-vis spectrophotometer (Shimadzu UV-1800, Kyoto, Japan). The radical scavenging activity (%) was calculated as follows:

(1) $$\begin{array}{c} \text{Scavenging activity }\left(\%\right)=\;\left[\frac{1-A_{1}}{A}\right]\times 100\%, \end{array}$$

where *A*_1_ is the absorbance of the sample, and *A*_0_ is the absorbance of the blank group.

### 2.4. TEAC Assay

The antioxidant activity against ABTS+ was measured as described by Strail et al. [51] with some modifications. ABTS+ was formed by reacting 7.0 mM ABTS with 4.95 mM potassium persulfate solution at room temperature in the dark for 12 h. Then, the solution was diluted with 80% ethanol before measuring the absorbance (0.70 ± 0.02) at 734 nm. After incubation of 20 *μ*L sample solution (0.2–4 mg/mL) with 3.0 mL ABTS+ at 30°C for 10 min, the absorbance was measured at 734 nm by the UV-vis spectrophotometer. The antioxidant capacity was expressed as mg of Trolox/g of samples (TEAC), and the TEAC was calculated as follows:

(2) $$\begin{array}{c} \text{TEAC}=\frac{\left(A_{0}-A_{1}\right)-a}{b}\times \frac{V}{W}, \end{array}$$

where *A*_1_ is the absorbance of the sample, *A*_0_ is the absorbance of the blank group, *b* is the slope of the standard curve, *a* is the intercept of the standard curve, *V* is the volume of Trolox (*μ*L), and *W* is the weight of extracts (g).

### 2.5. FRAP

The antioxidant activity against Fe^3+^-TPTZ was performed according to the method established by Benzie et al. [52] with some modifications. Fresh FRAP reagent was prepared by mixing 300 mm acetate buffer (pH 3.6), 10 mm TPTZ solution, and 20 mm FeCl_3_·6H_2_O solution at the ratio of 10 : 1:1. A volume of 3.0 mL FRAP reagent was added to 20 L sample solution (0.2–4 mg/mL) and incubated at 37°C for 30 min. The absorbance of the reaction mixture was measured at 593 nm. FRAP was expressed as the number of moles of FeSO_4_ required to achieve the same absorbance per g of samples and calculated as follows:

(3) $$\begin{array}{c} \text{FRAP}=\frac{\left[A_{1}-\;A_{0}\right]-a}{b}\times \frac{1}{W}, \end{array}$$

where *A*_1_ is the absorbance of the sample, *A*_0_ is the absorbance of the blank group, *b* is the slope of the standard curve, *a* is the intercept of the standard curve, and *W* is the weight of extracts (g).

### 2.6. Hydroxyl Radical Scavenging Assay

The hydroxyl radical scavenging capacity was evaluated according to the method described by Jin et al. [53] with some modifications. Briefly, 1.0 mL phosphate-buffered saline (PBS, pH 7.4) was mixed with 0.5 mL (0.148 mg/mL) o-phenanthrene solution, 0.5 mL (0.208 mg/mL) ferrous sulfate solution, and 1.0 mL sample solution (0.2–4 mg/mL). After the mixture was vortexed, 1.0 mL (0.01%) hydrogen peroxide (H_2_O_2_) solution was added and incubated at 37°C for 60 min. The absorbance was measured at 536 nm. The hydroxyl radical scavenging capacity was calculated as follows:

(4) $$\begin{array}{c} \text{Scavenging activity}\left(\%\right)=\left[\frac{\left(A_{1}-A_{0}\right)}{\left(A_{2}-A_{0}\right)}\right]\times 100\%, \end{array}$$

where *A*_1_ is the absorbance of the sample, *A*_0_ is the absorbance of the blank control group, and *A*_2_ is the absorbance of the sample and reagent blank group.

### 2.7. HPLC Conditions

Herein, we used traditional methods to select the extract with high antioxidant activity, and the antioxidant components were screened using the HPLC-DPPH scavenging method and HPLC-hydroxyl radical scavenging method [54]. The chromatography selected optimal chromatographic conditions to separate the components in the extract. Then, the content of the sample components before and after the addition of the free radicals was detected using a UV detector. Next, the components with antioxidant capacity were selected by comparing the changes in the peak area or peak height of the corresponding components in the two chromatograms. Antioxidant components in the extract with high oxidation activity were screened by using an Agilent HPLC system with a photodiode-array detector (1260 series, Santa Clara, CA, USA). The chromatographic separation was carried out using a Shimadzu VP-ODS C18 (150 × 4.6 mm, 5 *μ*m) with solvent A (0.1% formic acid) and solvent B (acetonitrile). The gradient condition was as follows: 0 min, 18% B; 0–5 min, 25% B; 5–10 min, 35% B; 10–15 min, 60% B; 15–20 min, 90% B; and 20–30 min, 18% B. The flow rate was 1.0 mL/min, the injection volume was 20 *μ*L, the column temperature was set at 30°C, and the detection wavelength was 254 nm.

### 2.8. HPLC-DPPH Scavenging Method

The majority of the phenolic antioxidants can quench DPPH radicals, and hence, DPPH radicals are often used to screen the antioxidant activities of phenolic compounds [55]. In this study, we used the HPLC-DPPH scavenging method to determine the peak area of each component in the sample before and after adding DPPH. The components of the reduced peak area harbored antioxidant activity. Briefly, 250 *μ*L sample solution (4 mg/L) and 250 *μ*L DPPH solution (500 *μ*M) were mixed and incubated at 37°C in the dark for 30 min. The mixture was then filtered through a 0.45 *μ*m membrane and determined according to the chromatographic conditions in 2.2.5.

### 2.9. HPLC-Hydroxyl Radical Scavenging Method

Briefly, 200 *μ*L H_2_O_2_ solution (25 mM) and 200 *μ*L of FeSO_4_ solution (250 *μ*M) were added to 250 *μ*L sample solution (4 mg/L). The mixture was incubated at room temperature in the dark for 7 min, filtered with 0.45 *μ*m membrane, and measured according to the chromatographic conditions in 2.2.5.

### 2.10. Identification of Active Antioxidant Ingredients

The identification experiment was carried out using an Agilent HPLC-Q-TOF LC/MS (1260 series, 6520 series) and an Agilent HPLC system with a photodiode array detector (1260 series). The chromatographic separation was conducted using a Extend Agilent C18 (50 × 2.1 mm, 1.8 *μ*m) with solvent A (0.1% formic acid) and solvent B (acetonitrile). The gradient condition was as follows: 0 min, 18% B; 0–5 min, 25% B; 5–10 min, 35% B; 10–15 min, 60% B; and 15–20 min, 90% B. The flow rate was 0.2 mL/min, injection volume was 1 *µ*L, the column temperature was room temperature, the scanning range was 50–1500, and the capillary voltage was 4000 V. Nitrogen was used as dry gas at a flow rate of 10 mL/min, the temperature was 350°C, and the atomizer pressure was 40 psi. The mass spectrogram and chromatogram were recorded under the negative ion source. After acquiring the molecular weight, the antioxidant components in the sample were further identified based on their standard products by HPLC, according to the chromatographic conditions in 2.2.5.

### 2.11. Statistical Analysis

All experimental data were expressed as mean ± standard deviation (SD) of three independent experiments in triplicate and analyzed by SPSS (version 17.0). One-way analysis of variance (ANOVA) and Tukey multiple comparisons were carried out to test any significant differences between the means. *P* < 0.05 indicated statistical significance.

## 3. Results

### 3.1. Screening of Antioxidant Extracts

#### 3.1.1. Comparison of DPPH Free Radical Scavenging Capacity of Extracts

**Figure 1:**
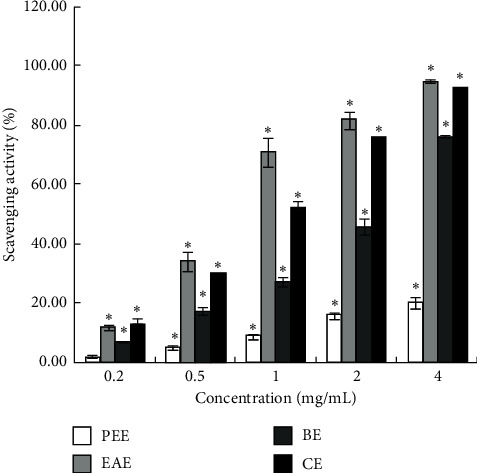
DPPH radical scavenging activity (%) of four extracts.  ^*∗*^*P* < 0.05 vs. the blank group.

**Figure 2:**
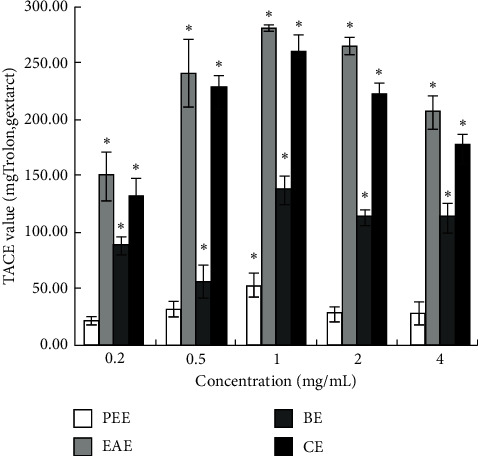
TEAC value (Trolox mg/g extract) of four extracts.  ^*∗*^*P* < 0.05 vs. the blank group.

DPPH has been widely used in the determination of antioxidant free radical scavenging capacity [56]. The scavenging rate of each extract is shown in Figure 1. Subsequently, the DPPH scavenging activity of EAE and CE was similar to the sample concentrations of 0.2 mg/mL (*P* > 0.05) and 4 mg/mL (*P* > 0.05), respectively, while DPPH scavenging activity of EAE and all the other extracts is the largest difference at the sample concentration of 1 mg/mL (*P* < 0.05). The DPPH scavenging activity of EAE was dose-dependent in the concentration range of 0.2–4 mg/mL and occurred in the following order: EAE > CE > BE > PEE (Figure 2).

#### 3.1.2. Comparison of ABTS+ Free Radical Scavenging Capacity of Extracts

The TEAC values of different concentrations of each extract are shown in Figure 2. In addition to PPE, the other extracts showed a robust ability to scavenge ABTS+ radicals. However, no significant difference was detected in the TEAC values between EAE and CE at the sample concentrations of 0.2, 0.5, and 1 mg/mL (*P* > 0.05). On the other hand, in the sample concentration range of 0.2–4 mg/mL, the TEAC values of EAE were 150.78 ± 20.94, 241.72 ± 30.34, 281.36 ± 3.17, 265.54 ± 7.79, and 207.59 ± 14.45 mg Trolox/g extract, respectively. The TEAC value was not dose-dependent because the weight of the extract (*W*) varied in the five different concentrations of each extract. If calculated according to formula (1), the antioxidant capacity was dose-dependent and expressed in terms of scavenging activity.

#### 3.1.3. Comparison of the Reducing Power of Extracts

**Figure 3:**
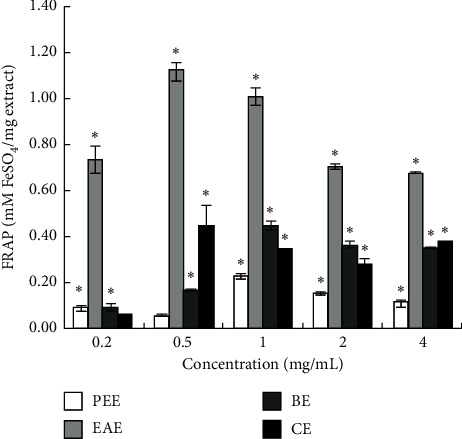
FRAP value (FeSO_4_ mM/mg extract) of four extracts.  ^*∗*^*P* < 0.05 vs. the blank group.

In this experiment, the reduction ability of CE and EAE was dissimilar (*P* < 0.05). At different concentrations, the reduced ability of EAE was significantly higher than that of other extracts, indicating a high reduction ability of Fe^3+^. In the concentration range of 0.2–4 mg/mL, the FRAP values of EAE were 0.74 ± 0.06, 1.12 ± 0.04, 1.02 ± 0.04, 0.71 ± 0.01, and 0.68 ± 0.00 mM FeSO_4_/g extract, respectively (Figure 3).

#### 3.1.4. Comparison of Hydroxyl Radicals Scavenging Capacity of Extracts

**Figure 4:**
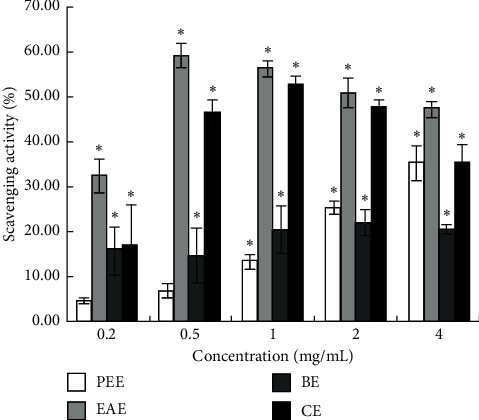
Hydroxyl radicals scavenging activity (%) of four extracts.  ^*∗*^*P* < 0.05 vs. the blank group.

Figure 4 shows the highest clearance rate of EAE, although the clearance rates of CE of 1 and 2 mg/mL concentrations are close to that of EAE (*P* > 0.05). At 0.2, 0.5, and 0.4 mg/mL, the clearance rate of EAE was significantly different from that of other extracts (*P* < 0.05). In the concentration range of 0.2–4 mg/mL, the scavenging activity of hydroxyl radicals in EAE was 32.69 ± 3.66%, 59.73 ± 2.76%, 56.80 ± 1.69%, 51.28 ± 3.47%, and 47.60 ± 1.63%, respectively.

### 3.2. Screening Antioxidant Components in the Extract with High Antioxidant Activity by HPLC

The present study demonstrated that the EAE had the strongest antioxidant activity among the four extracts. Then, we used HPLC to analyze and screen the antioxidant components by HPLC-DPPH and HPLC-hydroxyl radical scavenging methods.

**Figure 5:**
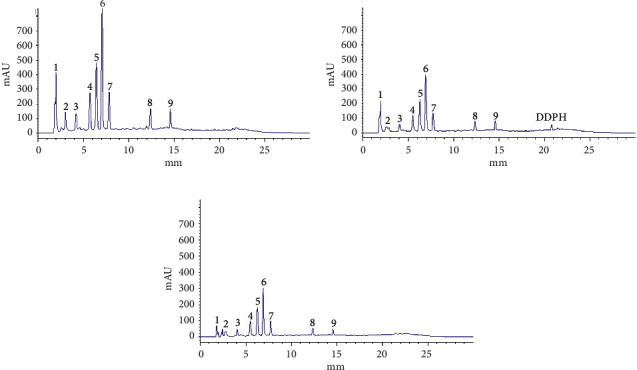
Chromatogram of antioxidant components in EAE (4 mg/mL) screened by HPLC. (a) Chromatogram of the sample without any added reagent. (b) Chromatogram of the sample with added DPPH. (c) Chromatogram of the sample with added hydroxyl radical.

When no reagent was added to the sample solution, nine chromatographic peaks were obtained (Figure 5(a)). After the DPPH solution (Figure 5(b)) or hydroxyl radical reagent (Figure 5(c)) was added to the sample solution, distinct changes were detected in the area and height of the nine peaks. Compared to the chromatograms of the sample before and after the reaction, the amount of free radicals and the antioxidant content decreased with the progress in the antioxidant reaction (Figure 5). A total of nine components were identified as peaks.

### 3.3. Identifying Antioxidant Components in the Extract with High Antioxidant Activity

**Figure 6:**
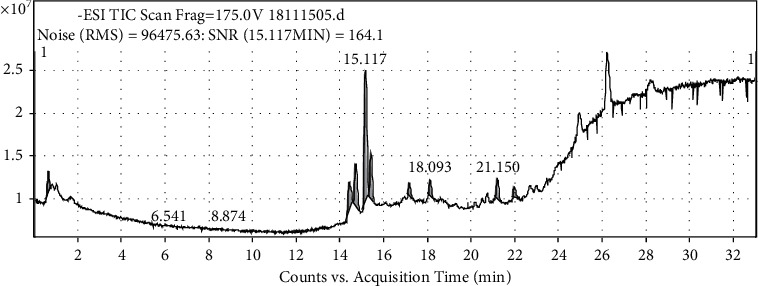
Total ion current diagram of EAE (4 mg/mL).

**Figure 7:**
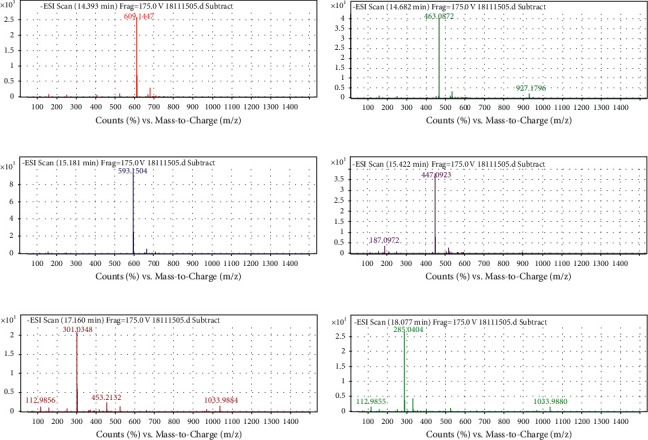
Mass chromatogram of components in EAE (4 mg/mL). (a) Mass chromatogram of rutin. (b) Mass chromatogram of isoquercetin. (c) Mass chromatogram of kaempferol-3-o-rutoside. (d) Mass chromatogram of astragaloside. (e) Mass chromatogram of quercetin. (f) Mass chromatogram of kaempferol.

**Figure 8:**
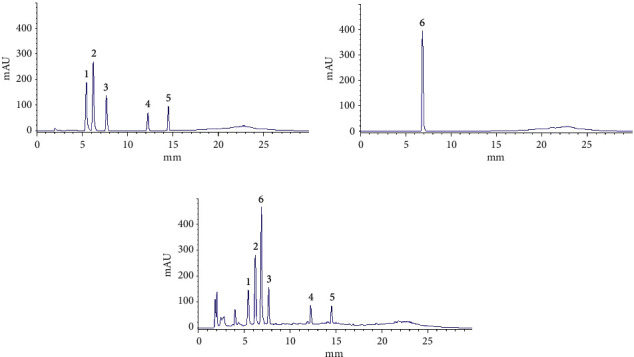
Chromatogram of standard reference and EAE (4 mg/mL). (a) Chromatogram of mixed standard reference. (b) Chromatogram of kaempferol-3-o-rutoside standard reference. (c) Chromatogram of EAE: 1, rutin; 2, isoquercetin; 3, astragaloside; 4, quercetin; 5, kaempferol; 6, kaempferol-3-o-rutoside.

The response of each component in the EAE extract to the negative ion source is better than that of the positive ion source, which is the same as reported previously [57, 58]. Based on the total ion chromatogram (Figure 6) and six-component mass spectrogram (Figure 7) under the negative ion detection mode and the molecular weight and molecular structure (Table 1) analyzed by the data of the workstation, we speculated the potential compounds and compared them with the corresponding standards by HPLC. Finally, we identified six compounds, namely, rutin, isoquercetin, astragaloside, quercetin, kaempferol, and kaempferol-3-o-rutoside (Figure 8).

## 4. Discussion

The current results are different from those of Sun et al. that 80% methanol extract of *Tetrastigma hemsleyanum* had better scavenging ability to DPPH and ABTS+ free radicals than ethyl acetate extract and hexane extract [49], which could be attributed to different extraction methods, origins of the herb, and concentrations of the samples. In this study, the total antioxidant capacity of CE was not equivalent to that of EAE, although the trend of CE in scavenging DPPH radicals, ABTS+ radicals, hydroxyl radicals, and reducing Fe^3+^ was consistent with that of EAE (Figures 1–4). This might be ascribed to the lower content of flavonoids in CE than that in EAE.

Figure 5 shows that the content of peaks 1, 2, and 3 also decreased; however, the molecular weight was not determined by HPLC-Q-TOF LC/MS. Furthermore, phenolic acids, such as catechin, epicatechin, gallic acid, procyanidin B_1_, and procyanidin B_2_ [59, 60], with strong antioxidant activity, have not been identified in EAE, CE, PEE, and BE, which might be due to the extraction process or the low content of phenolic acids in the extracts. The antioxidative activity of EAE in vivo and the distribution and metabolism of each antioxidant component in the organisms are not clear, and the mechanism of EAE underlying the antioxidative effect remains to be elucidated.

The six identified compounds were flavonoids and polyhydroxy compounds, and their antioxidant activity has been reported. Among these, rutin is a strong antioxidant [57], and its antioxidant activity is based on three mechanisms: the o-dihydroxy structure in B ring, the 2,3 double bond of the 4-oxo-functional group coupling in C ring, and the 5-OH and 7-OH groups of the 4-oxo-functional group in A and C rings. Rutin alleviates early brain injury of rats with subarachnoid hemorrhage by antioxidation and antiapoptosis [58]. Quercetin inhibits the activities of acetylcholinesterase and butyryl cholinesterase and Fe^2+^ induces lipid peroxidation in the brain homogenates of the rat, which are the putative mechanisms that underlie the quercetin-mediated management of oxidative stress-induced neurodegeneration [61]. Isoquercetin, a potent antioxidative compound, has neuroprotective capacities that are beneficial for the treatment of ischemic stroke and related diseases [62]. Astragalin, a major flavonoid, is a potential beneficial agent that protects the diabetic-induced spermatogenic dysfunction in male mice by increasing the antioxidant enzyme activities and inhibiting inflammation [63, 64]. Kaempferol and kaempferol-3-o-rutoside increases the DPPH and ABTS free radical scavenging activity and inhibits the T cell proliferation induced by concanavalin A and nitric oxide (NO) or reactive oxygen species (ROS) production in LPS-induced 264.7 macrophages cells [65].

## 5. Conclusions

In this study, for the first time, we used in vitro traditional antioxidant tests (DPPH assay, TEAC assay, FRAP, and hydroxyl radical scavenging assay) combined with HPLC-DPPH and HPLC-hydroxyl radical scavenging methods to screen out EAE with high antioxidant activity from *Tetrastigma hemsleyanum* and identified six antioxidant components by Q-TOF LC/MS and HPLC. The EAE may be used in food, medicine, and other fields to prevent the disease and assist the treatment of some oxidative stress-related diseases as these six compounds have significant biological activities. However, further studies are needed on the correlation between the content of each antioxidant component in EAE and the antioxidant capacity of EAE and the antioxidant mechanism.

## Figures and Tables

**Table 1:** Molecular weight and formula of six chemical components in EAE (4 mg/mL).

No.	tR/min	Molecular formula	Measured value (*m/z*)	Theoretical value (*m/z*)	Relative error (ppm)
1	14.393	C_27_H_30_O_16_	610.1494	610.1473	2.31
2	14.682	C_21_H_19_O_12_	464.0955	464.0944	2.24
3	15.181	C_27_H_29_O_15_	594.1585	594.1576	1.41
4	15.422	C_21_H_19_O_11_	448.1006	448.0996	2.19
5	17.160	C_15_H_9_O_7_	302.0427	302.0421	1.82
6	18.077	C_15_H_9_O_6_	286.0477	286.0477	0.09

## Data Availability

The datasets generated and analyzed during the present study are available from the corresponding author upon request.

## Abbreviations

DPPH: 1,1-Diphenyl-2-picrylhydrazyl radical, 2,2-diphenyl-1-(2,4,6-trinitrophenyl)hydrazyl
TEAC: Trolox equivalent antioxidant capacity
FRAP: Ferric reducing antioxidant power
ABTS: 2, 2'-Azino-bis(3-ethylbenzothiazoline-6-sulfonic acid)
HPLC: High-performance liquid chromatography
HPLC-Q-TOF LC/MS: High-performance liquid chromatography coupled with quadrupole time-of-flight tandem mass spectrometry
EAE: The ethyl acetate extract
CE: The crude extract
PEE: Petroleum ether extract
BE: n-Butanol extract
BHT: Dibutyl hydroxytoluene
BHA: Butyl hydroxyanisole
PG: Propyl gallate
TPTZ: 2,4,6-Tri-2-pyridinyl-1,3,5-triazine
Trolox: 6-Hydroxy-2,5,7,8-tetramethylchroman-2-carboxylic acid.
